# Lung Inflammation Resolution by RvD1 and RvD2 in a Receptor-Dependent Manner

**DOI:** 10.3390/pharmaceutics15051527

**Published:** 2023-05-18

**Authors:** Jin Gao, Yujie Su, Zhenjia Wang

**Affiliations:** Department of Pharmaceutical Sciences, College of Pharmacy and Pharmaceutical Sciences, Washington State University, Spokane, WA 99210, USA

**Keywords:** neutrophils, RvD1, RvD2, acute lung inflammation (ALI), inflammation resolution, GPR18, ALX/GPR32

## Abstract

Inflammation resolution is an active process via specialized pro-resolving mediators (SPMs) to fight invading microbes and repair tissue injury. RvD1 and RvD2 are SPMs produced from DHA during inflammation responses and show a benefit in treating inflammation disorders, but it is not completely understood how they act on vasculature and immune cells in the lung to promote inflammation resolution programs. Here, we studied how RvD1 and RvD2 regulated the interactions between endothelial cells and neutrophils in vitro and in vivo. In an acute lung inflammation (ALI) mouse model, we found that RvD1 and RvD2 resolved lung inflammation via their receptors (ALX/GPR32 or GPR18) and enhanced the macrophage phagocytosis of apoptotic neutrophils, which may be the molecular mechanism of lung inflammation resolution. Interestingly, we observed the higher potency of RvD1 over RvD2, which may be associated with unique downstream signaling pathways. Together, our studies suggest that the targeted delivery of these SPMs into inflammatory sites may be novel strategies with which to treat a wide range of inflammatory diseases.

## 1. Introduction

It is well known that the resolution of acute inflammation is a highly coordinated and physiologically active event [1]. During inflammation resolution, unsaturated fatty acids at the site of inflammation produce specialized pro-resolving mediators (SPMs) to control the inflammation progression. For example, arachidonic acid (C20:4) is metabolized to form lipoxins, and omega-3 polyunsaturated fatty acids (such as docosahexaenoic acid (DHA, C22:6) or eicosapentaenoic acid (EPA, C20:5)) produce a series of resolvins as well as protectins [2]. The uptake of omega-3 polyunsaturated fatty acids can manage inflammation responses and repairs injured tissues [3,4], which is associated with the enzymatical conversion of DHA and EPA into resolvins and protectins [5]. The molecular mechanisms of inflammation resolution have been investigated [1,6]. DHA can be metabolized by a lipoxygenase to produce a series of resolvins D (RvDs) [7,8,9]. For example, RvDs bind to GPCR receptors, initiating downstream signaling to regulate cellular functions. RvD1 binds to ALX/GPR32 receptors, highly expressed on human phagocytes, in order to increase their phagocytosis [7]. In addition, RvD1 limits mouse neutrophil recruitment to inflammatory loci via the binding of RvD1 to ALX receptors [10,11,12]. GPR18 is a receptor of RvD2, and RvD2 can prevent infections as well as protect against organ damage [11,13].

To effectively deliver RvD1 and RvD2 to inflammatory sites, cell-membrane-formed nanovesicles are utilized to target inflammatory tissues [12,14]. Using this approach, RvD1 and RvD2 can improve the therapies in acute respiratory distress syndrome (ARDS) and stroke. Studies demonstrate the therapeutic value of SPMs in treating a wide range of inflammatory diseases [15]; however, the molecular mechanisms through which RvD1 and RvD2 regulate inflammation resolution in order to prevent inflammatory responses are not well studied. In particular, it is not clear how RvDs control neutrophil adhesion and transmigration across vasculature, or how neutrophil apoptosis and phagocytosis are regulated by RvDs. It is not clear whether RvD1 and RvD2 have different potencies with which to resolve inflammation. 

Herein, we investigated the role of the receptors of RvD1 and RvD2 in regulating the adhesion and transmigration of neutrophils across endothelial cells in vitro and LPS-induced inflammation mouse models. In addition, we addressed how RvD1 or RvD2 enhanced neutrophil apoptosis and the macrophage phagocytosis of apoptotic neutrophils. In an acute lung inflammation mouse model, we demonstrated that RvD1 or RvD2 resolved inflammation responses via their GPCR receptors, thus preventing acute lung injury. Our studies reveal that the effective delivery of RvD1 or RvD2 to inflammatory sites will be a valuable therapeutic in treating inflammatory diseases.

## 2. Materials and Methods

### 2.1. Materials

RvD1 and RvD2 (10012554, 10007279) were purchased from Cayman (Ann Arbor, MI, USA). Dimethyl sulfoxide (D4540, DMSO), lipopolysaccharide (derived from O55:B5, L2880, LPS), Triton X-100, and polyformaldehyde (158127, PFA) were purchased from Sigma Chemical Co. (St. Louis, MO, USA). Rabbit anti-GPR18 polyclonal antibody (SAB4501252) was provided by Sigma (St. Louis, MO, USA). Rabbit anti-GPR32 polyclonal antibody was purchased from Raybiotech (14464050, Peachtree Corners, GA, USA). Rabbit anti-ALX polyclonal antibody was purchased from Invitrogen (720293, Eugene, OR, USA). Unless otherwise specified, the primary antibodies and the horse radish peroxidase (HRP)-labeled secondary antibodies were purchased from Santa Cruz Biotechnologies (Santa Cruz, CA, USA). Unless otherwise specified, the fluorescent antibodies for cell population identification were bought from Biolegend (San Diego, CA, USA).

### 2.2. Cell Culture

The HL60 cell line was purchased from ATCC (Manassas, VA, USA). The cells were cultured in an RPMI1640 medium (10041CV, Corning, NY, USA) supplemented with 10% fetal bovine serum (S11550H, Atlanta Biologicals, Flowery, GA, USA). Generally, the cells will be differentiated using 1.25 (*v*/*v*) DMSO for 4 days in order to generate neutrophil-like cells. Human umbilical vein endothelial cells (HUVECs) were purchased from Lonza (Walkersville, MD, USA). The cells were cultured in an endothelial-cell-growth-based medium supplemented with a serum kit (CCM027, R&D Systems, Minneapolis, MN, USA). The cells were typically activated by recombinant human TNF-α (570104, Biolegend, San Diego, CA, USA), 50 ng/mL for 4 h.

### 2.3. Animals

CD-1 mice weighing 25–30 g were purchased from Envigo Inc., New Jersey, NJ, USA. All mice were housed under specific pathogen-free conditions within the facilities of the Washington State University Spokane campus, and all animal studies were approved by the Institutional Animal Care and Use Committee of Washington State University (ASAF6063).

### 2.4. Cell Surface Marker Detection via Western Blotting

HUVECs were seeded at 2 × 10^6^/well in 3.5-inch plates one day ahead of performing the assay. The cells were treated with TNF-α (50 ng/mL), TNF-α (50 ng/mL) plus RvD1 (50 ng/mL), and TNF-α (50 ng/mL) plus RvD2 (50 ng/mL) for 4 h. The HUVECs were treated with the same volumes of vehicles as a control. The HUVECs were washed and lysed using 0.2 mL cell lysis buffer (Thermo Scientific, Rockford, IL, USA) and loaded on 12% SDS-PAGE for electrophoresis after the samples were boiled for 5 min with 50 µL at 5 times the loading buffer. We then transferred proteins in SDS-PAGE to a polyvinylidene fluoride (PVDF) membrane through using the wet membrane transfer method, as described previously [16,17]. Proteins were blotted using anti-ICAM-1, anti-VCAM-1, anti-integrin β1, anti-P-selectin, anti-L-selectin, anti-E-selectin, anti-PECAM-1, and anti-VE-cadherin monoclonal antibodies. GAPDH was detected through using anti-GAPDH antibodies as an internal reference. The Super Signal West Femto Maximum Sensitivity Substrate (34095, Thermo Scientific, Rockford, IL, USA) was used to visualize blot bands. The gray intensity of Western blots was obtained by Image Lab software (Bio-Rad, Hercules, CA, USA).

### 2.5. In Vitro Adhesion Assay

HUVECs were seeded at 5 × 10^5^/well in 6-well plates one day ahead of performing the assay. The cells were treated with TNF-α (50 ng/mL), TNF-α (50 ng/mL) plus RvD1 (50 ng/mL), and TNF-α (50 ng/mL) plus RvD2 (50 ng/mL) for 4 h. The HUVECs were treated with the same volumes of vehicles as a control. HUVECs were washed once with sterile PBS and incubated with 10^5^ differentiated HL60 cells (DMSO, 1.25% *v*/*v*, 4 days) pre-stained with DiD lipid dye (V22887, Invitrogen, Eugene, OR, USA) at 37 °C for 15 min. Thereafter, the cells were washed with PBS 3 times before the images were taken using a confocal microscope (A1 Plus, Nikon, Japan) with a water lens. The adhesion was analyzed by counting individual adhered cells per field, and the experiments were performed in triplicate.

### 2.6. Transmigration

The experiment was performed following the previously established method, with modifications [12]. Transwells (12 mm diameter membrane with pores 3 μm in diameter) were first coated with 0.2% gelatin for 0.5 h at room temperature. The HUVECs were added (10^4^ cells/well) to the transwells and cultured in a 24-well plate until the cells formed 100% confluence, observed under a microscope. Cytokines and chemokines (10 ng/mL fMLP, 10 ng/mL TNF-α, 50 ng/mL IL-1β, and 10 ng/mL IL-6) in 0.6 mL were added to the bottom wells. In 0.2 mL of cell culture medium, 10^5^ activated HL60 cells, along with RvD1 (50 ng/mL) or RvD2 (50 ng/mL), were placed in the upper chamber. Transmigration was performed for 3 h at 37 °C. Cells were collected from the bottom chamber, and they were gently washed with a PBS buffer, followed by the counting of cell numbers.

### 2.7. Cell Surface Molecules Detected by Flow Cytometry

To detect the adherent molecules, including integrin β_2_, L-selectin, integrin α_v_ and PSGL-1, ALX (RvD1 receptor), and GPR18 (RvD2 receptor) on resting or activated neutrophils, mice were challenged with 15 mg/kg LPS via an intraperitoneal injection followed by the intravenous administration of RvD1 (100 ng) or RvD2 (100 ng). The heparin-anticoagulated blood samples (around 5–10 µL) were centrifugated, and PBS containing 3% BSA was used to resuspend the pelleted cells. Then, 2 µg of rabbit anti-ALX polyclonal antibodies or rabbit anti-GPR18 polyclonal antibodies was added, followed by shaking for 30 min. The cells were collected through centrifugation and resuspended in PBS supplemented with 3% BSA; DyLight488-Donkey-anti-rabbit IgG, PE-DAZZLE-CD11b, PE/CY7-LY-6C, and Alexa Fluor 647-LY-6G (2 µg) were added, followed by shaking for 30 min, after which the cells were filtrated with 40 µm strainers before performing flow cytometry. The cells were analyzed via flow cytometry to identify neutrophils (CD11b^+^ Ly-6C^mid^ Ly-6G^+^).

### 2.8. Neutrophil Apoptosis

Heparin-anticoagulated blood samples were centrifugated in order to obtain the pellets. Then, PBS containing 2 mM CaCl2 was used to resuspend the pelleted cells; 5 µL Alexa 488-Annexin V (V13241, Invitrogen, Eugene, OR, USA) was added, followed by shaking for 30 min. PE-DAZZLE-CD11b, PE/CY7-LY-6C, and Alexa Fluor 647-LY-6G (2 µg) were then stained for the identification of neutrophils. The cells were then collected via centrifugation and resuspended in PBS containing 0.1% BSA. The cells were analyzed through flow cytometry in order to identify neutrophils (CD11b^+^ Ly-6C^low^ Ly-6G^+^), and the percentages of apoptotic cells were then analyzed.

### 2.9. In Vivo Phagocytosis of Apoptotic Neutrophils via Macrophages

Mice were administered LPS (15 mg/kg) via the intratracheal route and, 3 h later, RvD1 or RvD2 were i.v. injected. Neutralizing antibodies of the receptors were i.v. administered at 2.5 h after LPS challenge. The bronchoalveolar lavage fluids (BALFs) were collected as described previously [18,19]. Briefly, the mouse trachea was cannulated, and 1 mL of HBSS was intratracheally infused as well as withdrawn in order to obtain lavage fluid at 12 h after treatment (15 h after LPS administration). The total blood cells in BALFs were isolated by centrifuging BALFs at 800 g for 5 min, and the pelleted cells were fixed by using 4% PFA for 20 min on ice. After this, 70% iced ethanol was then applied to permeabilize the membrane. After being washed with PBS, the cells were stained with Alexa Fluor 488-anti-F4/80 and Alexa Fluor 488-anti-LY-6G for 30 min, with shaking. The stained cells were collected via centrifugation and resuspended in PBS containing 0.1% BSA, followed by an immediate analysis on a flow cytometer (Gallios, Beckman coulter, Indianapolis, IN, USA). The results were confirmed via confocal imaging (A1 Plus, Nikon, Japan).

### 2.10. Acute Lung Inflammation Model and Its Therapy

Mice were administered LPS (15 mg/kg) via the intratracheal route and, 3 h later, 100 ng RvD1 or RvD2 per animal was i.v. administered. For receptor-neutralizing groups, anti-ALX or anti-GPR18 antibodies at 0.5 mg/kg were i.v. administered 0.5 h before the injection of RvD1 and RvD2. The blood was collected via facial veins. BALFs and the lung organs were harvested for immunohistology after the mice were euthanized. The cell counting in BALFs was performed under a microscope, and the ratio of neutrophils was determined through flow cytometry (Galious, Beckman Coulter, Indianapolis, IN, USA). The cytokines in BALFs were measured via enzyme-linked immunosorbent assay (ELISA) kits from Biolegend (430904, 432604, and 431304, San Diego, CA, USA). 

### 2.11. Histology and Immunofluorescence

The lung tissues were collected from mice anesthetized with ketamine/xylazine (100:5 mg/kg), followed by being fixed in 4% PFA overnight at 4 °C and then sent to the Research Service Center Washington State University Spokane (Spokane, WA, USA) for paraffin embedding, sectioning, H&E staining, and imaging. For immunofluorescence experiments, tissue sections (5 µm thick) were deparaffinized and subjected to antigen retrieval by being incubated in 10 mM sodium citrate (pH 6.5) at 100 °C for 10 min. The tissue sections were then blocked in 3% rat serum (diluted in PBS containing 0.1% Triton X-100) for 1 h. The sections were washed with PBS three times and incubated with Alexa 488-anti-ICAM-1 overnight at 4 °C. Slides were washed three times in PBS with shaking. Finally, the slides were allowed to air dry and were stained with a FluoLast_TM_ mounting medium containing DAPI (1213, BioVision, Milpitas, CA, USA). Images were taken via the use of a confocal microscope (A1 Plus, Nikon, Japan). 

### 2.12. Statistical Analysis

Data are presented as the mean ± SDs. The statistical analyses were performed via the use of Origin software and a non-parametric one-way ANOVA. The differences between groups were significant at *p* ≤ 0.05, the differences between groups were considered very significant at *p* ≤ 0.01, and the differences between groups were considered extremely significant at *p* ≤ 0.001. 

## 3. Results

### 3.1. RvD1 or RvD2 Inhibits Leukocyte Adhering and Transmigration via Downregulating the Adherent Molecules of HUVECs 

**Figure 1:**
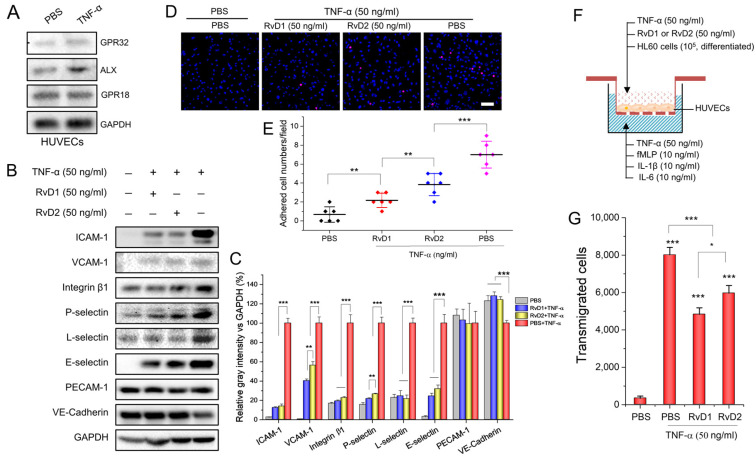
RvD1 and RvD2 regulate the expression of adhesion molecules in HUVECs and inhibit leukocyte adherence as well as transmigration in vitro. (**A**) The receptors of RvD1 and RvD2 expressed on HUVECs 4 h after being treated with 50 ng/mL TNF-α. (**B**) RvD1 and RvD2 downregulate adhesion molecules of HUVECs, and their quantification (**C**). (**D**) Confocal images show the adhesion of HL-60 to HUVECs after treatment with RvD1 or RvD2. A 100% confluence of HUVECs were treated with 50 ng/mL TNF-α for 4 h and incubated with 10^5^ neutrophil-like HL60 cells fluorescently labeled with DiD. (**E**) The quantification of confocal images in (**D**). (**F**) Transwell assay to measure the transmigration of neutrophils (HL60 cells) across confluent HUVECs and (**G**) the quantification results of (**F**). The data are expressed as means ± SD, n = 3–6. A one-way ANOVA was applied to determine the difference among multiple groups with * *p* ≤ 0.05, ** *p* ≤ 0.01, and *** *p* ≤ 0.001.

During the inflammation response, interactions between leukocytes and endothelium play a central role, and their interactions are regulated by adhesion molecules on both cells. First, we studied the role of endothelium in regulating the adhesion and transmigration of leukocytes in vitro. To determine whether GPR32, ALX, and GPR18 receptors are expressed by HUVECs, we performed a Western blot experiment and found that HUVECs expressed all of the above receptors, either before or after the cells were activated by 50 ng/mL TNF-α (Figure 1A). Next, we measured the expression of adhesion proteins and intercellular junction proteins on HUVECs before and after HUVECs were treated with 50 ng/mL TNF-α for 4 h. We found that most of the adhesion molecules were upregulated after TNF-α treatment; however, either RvD1 or RvD2 significantly inhibited the upregulation of these molecules. Interestingly, the downregulation of a tight-junction-related-protein, VE-cadherin, was shown when HUVECs were treated with TNF-α; however, RvD1 or RvD2 increased the expression of VE-cadherin (Figure 1B,C). The results demonstrate that RvD1 and RvD2 decreased leukocyte adhesion proteins and tight conjunction proteins (VE-cadherin) on HUEVCs; thus, they may inhibit leukocyte adhesion to endothelial cells and transmigration. Therefore, we conducted an in vitro adhesion assay to validate leukocyte endothelial adhesion. We used differentiated HL60 cells because they are similar to neutrophils after HL60 cells with 1.25% DMSO for 4 days [20,21]. This is consistent with our results, which showed that HL60 cells expressed various adhesion molecules as native neutrophils; thus, HL60 cells can be used for studies on interactions between neutrophils and endothelial cells (Figure S1). As shown in Figure 1D,E, when RvD1 or RvD2 was present there were less neutrophil-like HL60 cells adhered on the inflamed HUVEC cell layer. Furthermore, we performed an in vitro transmigration assay and the results showed that either RvD1 or RvD2 inhibited neutrophil-like HL60 cell transmigration significantly (Figure 1F,G), which is in line with previous findings [22,23]. The result suggests that RvD1 or RvD2 may effectively alleviate leukocyte transmigration across the blood vessel endothelial barrier. 

### 3.2. RvD1 or RvD2 Downregulates the Adhesion Proteins of Neutrophils and Promotes Neutrophil Apoptosis

**Figure 2:**
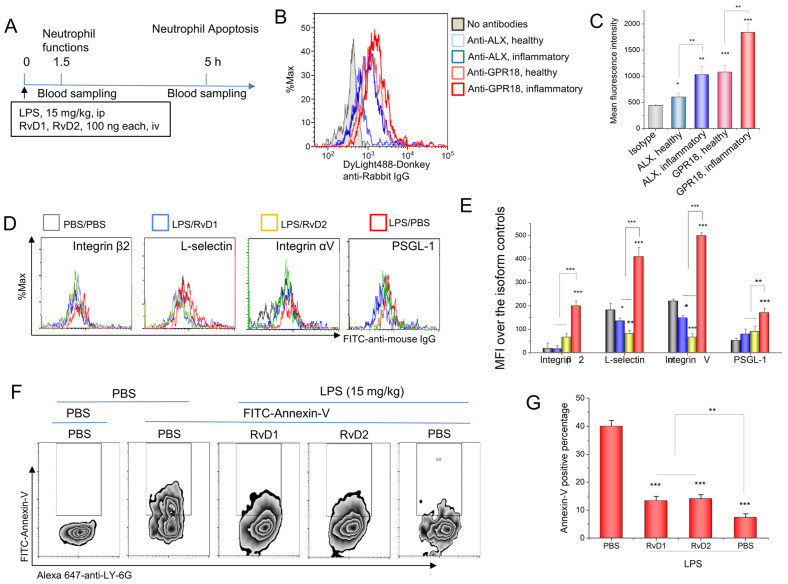
RvD1 and RvD2 attenuate the expression of neutrophil adhesion molecules and increase neutrophil apoptosis in vivo. (**A**) The animal protocol for analyzing neutrophil functions after the treatment with RvD1 and RvD2. (**B**) The expression of RvD1 and RvD2 receptors in mouse neutrophils via flow cytometry, and their quantitative analysis (**C**). (**D**) Adhesion molecules on neutrophils regulated by RvD1 and RvD2, and their quantification (**E**). (**F**) RvD1 and RvD2 promote the apoptosis of inflammatory neutrophils in mouse blood 5 h after LPS challenge of a 15 mg/kg i.p. injection, and their quantitative analysis (**G**). The data are expressed as the mean ± SDs, n = 3. A one-way ANOVA was applied to determine the difference among multiple groups, with * *p* ≤ 0.05, ** *p* ≤ 0.01, and *** *p* ≤ 0.001.

To address whether RvD1 or RvD2 regulates neutrophil functions during the inflammatory response, we measured the expression of RvD1 or RvD2 receptors on neutrophils. ALX (a receptor for RvD1) and GPR18 (a receptor for RvD2) are expressed in mice [24]; this being the case, we set up in vivo experiments to measure the receptor expression on neutrophils via the use of flow cytometry. To measure the expression of adhesion molecules on activated neutrophils, we created an inflammatory mouse model through the i.p. administration of 15 mg/kg LPS (Figure 2A). At a time of 1.5 h after LPS challenging mice, we analyzed the RvD1 receptor (ALX) and RvD2 receptor (GPR18) via the use of flow cytometry. The results demonstrated that these receptors were expressed on resting neutrophils, and that they increased when neutrophils were activated (Figure 2B,C). Next, we asked whether the treatment of RvD1 and RvD2 to mice can regulate neutrophil functions in vivo. Following the protocol shown in Figure 2A, we measured the expression of adhesion molecules on neutrophils, including integrin β2, L-selectin, integrin αV, and PSGL-1, and we found that RvD1 or RvD2 attenuated the expression of adhesion molecules increased by LPS (Figure 2D,E). Neutrophil apoptosis is one of the molecular mechanisms with which to resolve inflammatory responses [1,25]. The externalization of phosphatidylserine (PS) on the cell membrane is a marker of cell apoptosis; this being the case, we stained the cells with annexin-V to determine the externalization of PS. The results in Figure 2F,G show that LPS induced a decrease in the staining of annexin-V on neutrophils compared to healthy mice, indicating that LPS prevented neutrophil apoptosis, thus increasing neutrophil adhesion and transmigration in order to possibly intensify inflammatory responses; however, the administration of RvD1 and RvD2 to LPS-challenged mice increased neutrophil apoptosis (Figure 2F,G). Together, the results on the downregulation of adhesion proteins on neutrophils and increased neutrophil apoptosis (Figure 2F,G) suggest that RvD1 and RvD2 could be novel therapeutic agents with which to treat inflammatory diseases.

### 3.3. RvD1 or RvD2 Prevent Inflammation in an Acute Lung Inflammatory Model via Their Receptors

**Figure 3:**
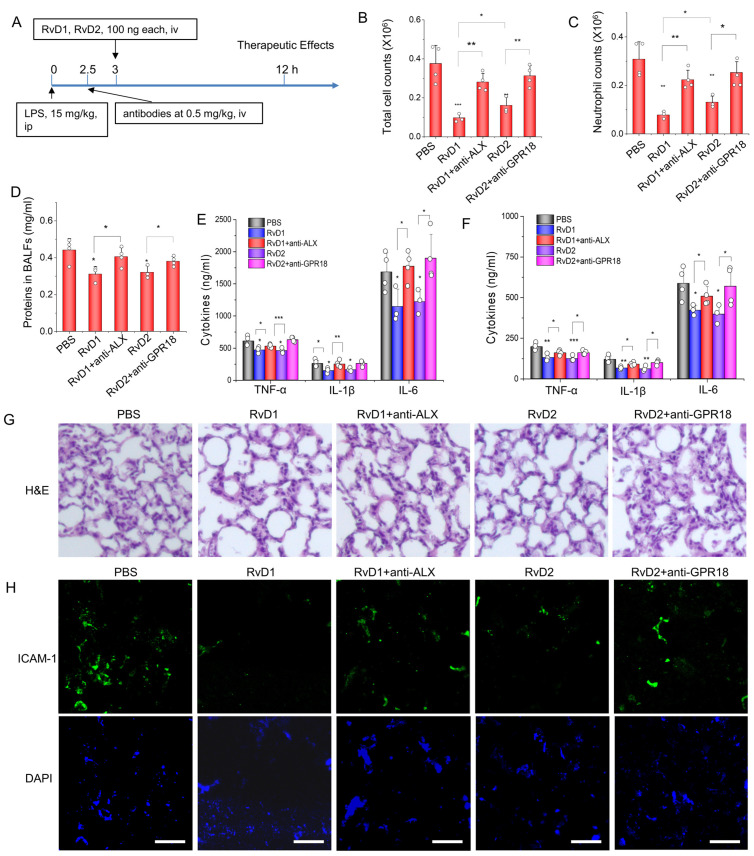
Lung inflammation resolution is dependent on RvD1 or RvD2 action on their receptors expressed on neutrophils and the endothelium in an acute lung inflammation model. (**A**) The animal experiment protocol with which to address therapeutic effects of RvD1 or RvD2. The animals were challenged with 15 mg/kg LPS and, 3 h later, the animals were treated with resolvins with or without the corresponding receptor antibodies. The animal blood, lung tissues, and BALFs were collected to measure inflammatory responses. Total cells (**B**) and neutrophils (**C**) in BALFs. (**D**) Protein contents in BALFs were determined via the BCA method. Cytokines, including TNF-α, IL-1β, and IL-6, in plasma (**E**) and BALFs (**F**) were determined by ELISA kits. (**G**) Typical H&E staining images of the lungs in each group. Magnification 100×. (**H**) Immunofluorescent images of ICAM-1 expression in the lung. Scale bar 100 µm. The data are expressed as the mean ± SDs, n = 3. A one-way ANOVA was applied to determine the difference among multiple groups, with * *p* ≤ 0.05, ** *p* ≤ 0.01, and *** *p* ≤ 0.001.

To address the therapeutic values of RvD1 and RvD2, we established an acute lung inflammation animal model, and studied whether the therapeutical effect of resolvins was dependent on their receptors (Figure 3A). At a time of 12 h after the animals were treated with RvD1 and RvD2, we measured the total cell number and neutrophil number in BALFs. As shown in Figure 3B,C, either lung-infiltrating cells or neutrophils were decreased when the mice were treated with resolvins; however, the administration of antibodies of RvD1 or RvD2 receptors to mice impaired the therapeutical effect of resolvins. Moreover, the proteins in BALFs also showed a similar trend, in which the therapeutic effect of resolvins was dependent on their receptors (Figure 3D), suggesting that RvD1 and RvD2 can repair the inflammation-induced leakiness of lung vasculature. Cytokines, including TNF-α, IL-1β, and IL-6, in blood or BALFs, were also measured. Similarly, RvD1 and RvD2 prevented the production of cytokines in circulation and the lungs, but the effect was impaired when the receptors of RvD1 and RvD2 were blocked by their antibodies (Figure 3E,F). We also studied the histological morphology of the mouse lung via the use of H&E, and found that either RvD1 or RvD2 can improve alveolar morphologies with less leukocyte infiltration; however, when the receptor antibodies were administered, the therapeutical effect of resolvins was diminished (Figure 3G). The infiltration of neutrophils in the lung is dependent on the activation of the endothelium [26]; this being the case, the lung sections were fluorescently stained with anti-ICAM-1. We observed that either RvD1 or RvD2 can significantly inhibit the increased expression of ICAM-1 on an inflammatory lung induced by LPS; however, the administration of receptor antibodies impaired the therapeutic effect of RvD1 or RvD2 (Figure 3H). Together, the data shown in Figure 3 demonstrate that RvD1 or RvD2 can act on their receptors on endothelial cells or leukocytes to promote inflammation resolution, thus mitigating lung damage and preventing lung edema due to an infection.

### 3.4. RvD1 or RvD2 Enhance the Phagocytosis of Macrophages 

**Figure 4:**
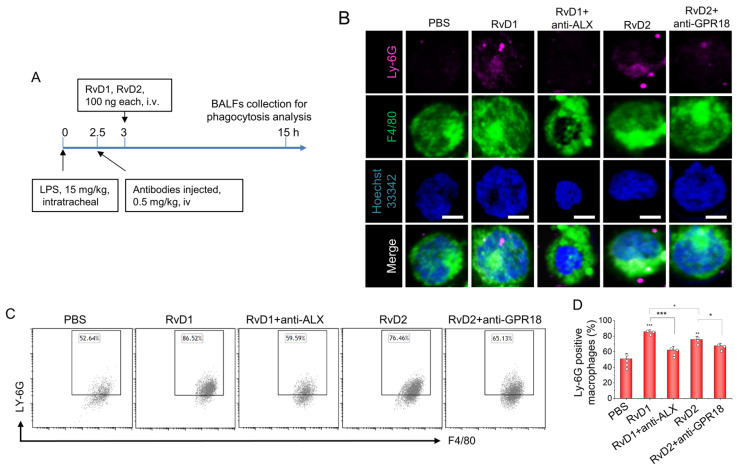
Phagocytosis of apoptotic neutrophils by macrophages is a mechanism of lung inflammation resolution in acute lung inflammation (ALI) induced by LPS. (**A**) The animal experiment protocol with which to address macrophage phagocytosis in the lungs. (**B**) At a time of 12 h after treatment with resolvins in ALI, macrophages in BALFs were collected and fixed. The anti-LY-6G antibody was used to identify neutrophils or related apoptotic bodies after macrophage membranes were permeabilized. The colocalization of LY-6G and F4/80 signals indicates the phagocytosis of apoptotic neutrophils by macrophages. Scale bar 10 µm. (**C**) To quantitatively analyze phagocytosis, flow cytometry was conducted to measure percentages of neutrophils or apoptotic bodies containing macrophages, and their quantitative analysis (**D**). The data are expressed as the mean ± SDs, n = 3. A one-way ANOVA was applied to determine the difference among multiple groups, with * *p* ≤ 0.05, ** *p* ≤ 0.01, and *** *p* ≤ 0.001.

Previous studies have proven that resolvins can reduce TNF-a–mediated inflammation in macrophages [27] and enhance the phagocytosis of apoptotic thymocytes by macrophages [28]. There are apoptotic neutrophils in inflammatory lungs during lung infections, so we hypothesized that the phagocytosis of neutrophils by macrophages could be a mechanism with which to resolve lung inflammation when we treated the mice with RvD1 or RvD2. Thereafter, we conducted an in vivo experiment and established a local acute lung inflammation animal model through intratracheal administration at 15 mg/kg LPS (Figure 4A). At 15 h after LPS challenge and 12 h after treatment with resolvins, the local macrophages in the lung were collected and observed under a fluorescent microscope. We found that, with or without the treatment of resolvins, macrophages phagocytosed some apoptotic neutrophils or apoptotic bodies, but the macrophages increased to take up more cells or bodies in the group treated with RvD1 and RvD2 (Figure 4B). To quantitatively analyze the phagocytosis of apoptotic neutrophils by macrophages, we conducted a flow cytometry assay and found that enhanced phagocytosis by macrophages can be abolished via the administration of receptor antibodies, indicating that the action is receptor-dependent (Figure 4C,D). Importantly, this is the first time that it has been demonstrated that resolvins can enhance lung inflammation resolution via the enhanced phagocytosis of apoptotic neutrophils by macrophages. 

## 4. Discussion

It is widely known that acute inflammation is a protective response to microbe invasion and tissue injury. Generally there are two phases: the inflammation phase and resolution phase [29]. Various physiological processes occur during acute inflammation, including changes in the blood vessel endothelial layer, adhesion molecule upregulation on leukocytes, increased cytokines, leukocyte infiltration, and tissue edema. To prevent an inflammation response that may cause tissue damage and chronic inflammation, nanotechnologies are used to effectively deliver drugs to inflammatory sites for managing inflammation scale [12,14,18,19,30,31,32,33,34,35,36,37,38,39,40]. 

Previous studies have demonstrated the specific binding of RvD2 to human recombinant GPR18 [9] and the downstream pathway of RvD2-GPR18 binding, including the phosphorylation of CREB, ERK1/2, and STAT3 [13]. In addition, it has recently been confirmed that receptors, such as the orphan receptor GPR32 and the ALX receptor, can bind RvD1 [7]. In particular, the binding of RvD1 to GPR32 was four times higher than that of the ALX receptor [10]. Resolvins are identified in inflammatory tissues via the use of HLPC–mass spectroscopy. For example, it was shown that resolvins derived from DHA can regulate neutrophil functions via activating their receptors to regulate downstream signaling [10]. RvD2 was reported to regulate leukocytes, control microbial sepsis, and reduce neutrophil–endothelium interaction via the nitric oxide (NO) pathway [8]. RvD1 can counteract vascular cell permeability induced by lipopolysaccharides (LPS).

HUVECs express the receptors of RvD1 and RvD2, and RvD1 shows the attenuation of endothelial vasculature permeability, neutrophil infiltration [41], and inflammation resolution [14,42]; however, there are a lack of studies that systemically investigate whether the actions of RvD1 or RvD2 are dependent on their receptors on both endothelial cells and neutrophils during inflammation. It is not clear how RvD1 or RvD2 regulate acute lung inflammation, nor what the molecular mechanism of inflammation resolution is in the lungs. 

**Figure 5:**
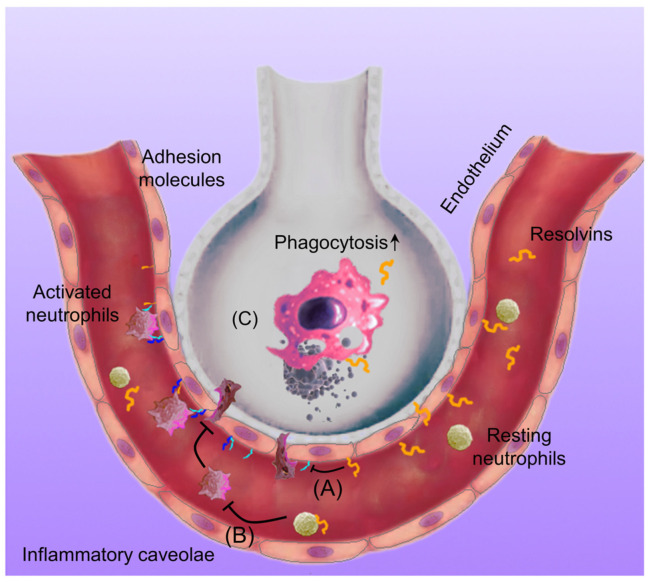
The molecular mechanism of RvD1/RvD2 in regulating the inflammation resolution in the lung. LPS induces an inflammatory response in the lungs, and endothelial cells as well as neutrophils upregulate their adhesion molecules to promote neutrophil adhesion and transmigration in the lungs. If the inflammation is out of control, it may cause tissue damage and chronic inflammation; however, when treating it with resolvins, the adhesion molecule expression on endothelial cells (A) and neutrophils (B) is alleviated, thus mitigating neutrophil transmigration and neutrophil apoptosis. Next, resolvins enhance the macrophage phagocytosis of apoptotic neutrophils (C), thus maintaining tissue homeostasis in the lungs.

In the present study, we systemically investigated how RvD1 and RvD2 regulate the interactions between neutrophils and endothelial cells in vitro and in vivo, finding that the binding of RvD1 and RvD2 to their receptors decreased the expression of adhesion molecules on neutrophils and endothelial cells. The blocking of their receptors via the use of antibodies impaired the actions of RvD1 and RvD2 in inflammation resolution. Furthermore, we found that RvD1 and RvD2 enhance neutrophil apoptosis. In an acute lung inflammation mouse model, we observed that RvD1 or RvD2 inhibited neutrophil lung infiltration and cytokine production and repaired the lung vasculature (Figure 3). To further investigate the molecular mechanism of lung inflammation resolution, we studied the macrophage phagocytosis of apoptotic neutrophils in the lung. We found that RvD1 or RvD2 enhanced the phagocytosis of apoptotic neutrophils (Figure 4). This may be the molecular mechanism of lung inflammation resolution. We propose a molecular mechanism of inflammation resolution in the lung, as shown in Figure 5.

Interestingly, we noticed the difference between the therapeutic effects of RvD1 and RvD2 in the inflammation responses. Figure 1E,G shows that RvD1 was more potent than RvD2 in the inhibition of neutrophil adhesion to endothelial cells and transmigration. This feature was also observed in the acute lung inflammation mouse model (Figure 3B,C). In addition, RvD1 was more efficient than RvD2 in the phagocytosis of apoptotic neutrophils in the acute lung inflammation model (Figure 4D). Together, the results indicate that RvD1 is more potent than RvD2 in inflammation resolution to maintain homeostasis. The expression of the RvD1 receptor (ALX/GPR32) was comparable to that of the RvD2 receptor (GPR18) on endothelial cells, as shown in Figure 1A. In addition, it looks as though ALX expression on neutrophils was lower than that of GPR18 (Figure 2C). The results suggest that the expression of resolvin receptors may not play a big role in the different therapeutic effect between RvD1 and RvD2. RvD1 triggers several agonist-dependent signal transduction processes, including protein kinase C (PKC) signaling, the phosphoinositide 3-kinase (PI3K)/protein kinase B (Akt) pathway, the mitogen-activated protein kinase (MAPK) pathway, the release of calcium, and the production of oxidants [43]. On the other hand, RvD2 increases the expression of TGF-β1 and regulates Akt/GSK-3β signaling [44,45]. The different signaling pathways may play a central role in the efficacy of RvD1 and RvD2 in regulating inflammation resolution. 

## 5. Conclusions

In summary, we elucidate the molecular mechanisms of inflammation resolution via the binding of RvD1 and RvD2 to their receptors, thus preventing acute lung inflammation and injury. We also find that the inflammation resolution process is dependent on the receptors. We observe that RvD1 is more potent than RvD2 in preventing a lung inflammation response, and that the difference may be associated with the downstream signaling pathways for each receptor. Our study suggests that it is necessary to develop novel delivery tools to effectively deliver RvD1 or RvD2 in order to treat a wide range of inflammatory diseases.

## Data Availability

The data presented in this study are available on request from the corresponding author.

## Supplementary Materials

The following supporting information can be downloaded at: https://www.mdpi.com/article/10.3390/pharmaceutics15051527/s1, Figure S1: The expression of the adherent molecules on the surface of HL60 cells; Figure S2: Gating strategy for circulating monocytes and neutrophils.
