# Supplementary material for: Lung Inflammation Resolution by RvD1 and RvD2 in a Receptor-Dependent Manner
Source: Pharmaceutics. 2023 May 18;15(5):1527. doi: 10.3390/pharmaceutics15051527 (PMC10221144; doi:10.3390/pharmaceutics15051527)
Supplement: Supplementary file 1 [file pharmaceutics-15-01527-s001.zip › pharmaceutics-2399537-supplementary.pdf]

Supplementary materials

Article

Lung inflammation resolution by RvD1 and RvD2 in a receptor dependent manner

Jin Gao, Yujie Su, and Zhenjia Wang *

Department of Pharmaceutical Sciences, College of Pharmacy and Pharmaceutical Sciences, Washington State University, Spokane, WA 99210, USA
***** Correspondence: zhenjia.wang@wsu.edu

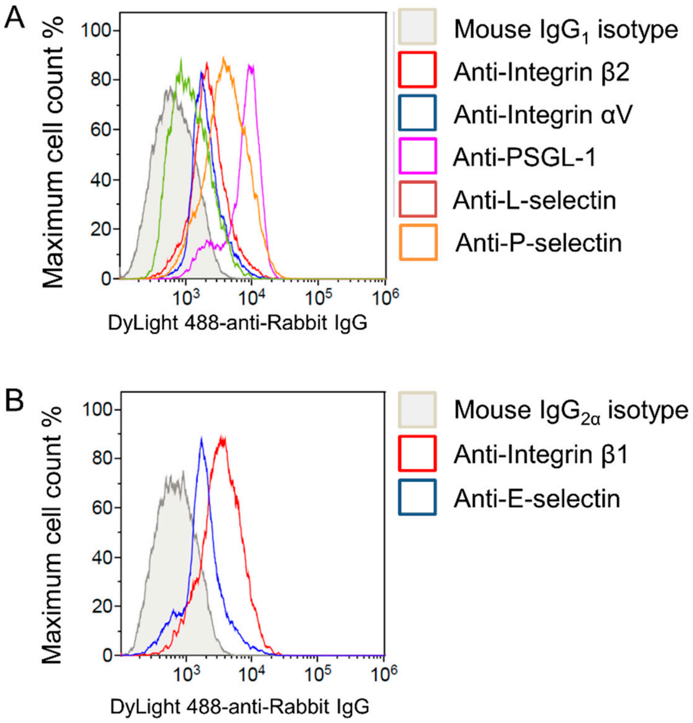

Figure S1. Expression of the adherent molecules on the surface of HL60 cells. HL60 cells were differentiated for 4 days with 1.25% (v/v) DMSO. Rabbit IgG1 isotype antibody (A) and Rabbit IgG2*α* isotype antibody (B) as negative controls for monoclonal antibodies.

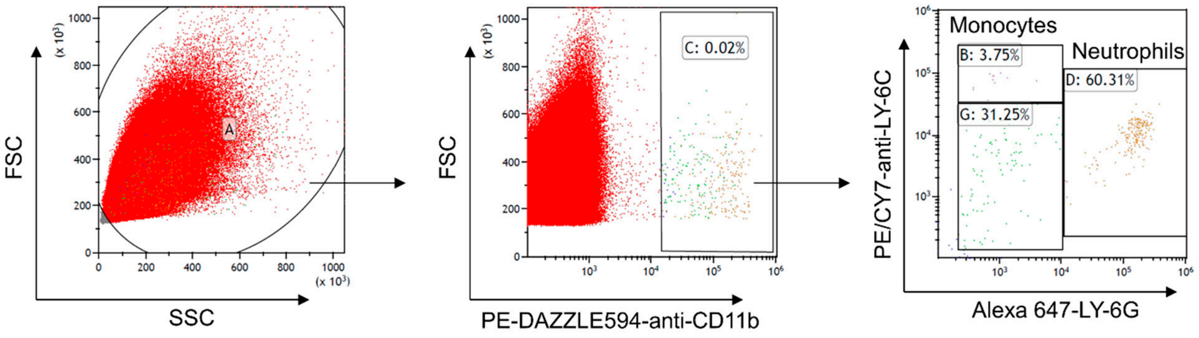

Figure S2. Gating strategy for circulating monocytes and neutrophils. Monocytes are populated as CD11b^{+}LY-6C^{high}LY-6G^{-}. Neutrophils are populated as CD11b^{+}LY-6C^{low}LY-6G^{+}.
